# Hospital Readmission of Adolescents and Young Adults With Complex Chronic Disease

**DOI:** 10.1001/jamanetworkopen.2019.7613

**Published:** 2019-07-24

**Authors:** Peter Dunbar, Matt Hall, James C. Gay, Clarissa Hoover, Jessica L. Markham, Jessica L. Bettenhausen, James M. Perrin, Karen A. Kuhlthau, Morgan Crossman, Brigid Garrity, Jay G. Berry

**Affiliations:** 1 Division of General Pediatrics, Boston Children’s Hospital, Harvard Medical School, Boston, Massachusetts; 2 Department of Pediatrics, Children’s Mercy Hospitals and Clinics, University of Missouri–Kansas City School of Medicine, Kansas City; 3 Children’s Hospital Association, Lenexa, Kansas; 4 Department of Pediatrics, Vanderbilt University Medical Center, Nashville, Tennessee; 5 Family Voices, Albuquerque, New Mexico; 6 Division of General Academic Pediatrics, Department of Pediatrics, MassGeneral Hospital for Children, Harvard Medical School, Boston, Massachusetts

## Abstract

**Importance:**

Adolescents and young adults (AYA) who have complex chronic disease (CCD) are a growing population that requires hospitalization to treat severe, acute health problems. These patients may have increased risk of readmission as demands on their self-management increase and as they transfer care from pediatric to adult health care practitioners.

**Objective:**

To assess variation across CCDs in the likelihood of readmission for AYA with increasing age.

**Design, Setting, and Participants:**

Retrospective 1-year cross-sectional study of the 2014 Agency for Healthcare Research and Quality Nationwide Readmissions Database for all US hospitals. Participants were 215 580 hospitalized individuals aged 15 to 30 years with cystic fibrosis (n = 15 213), type 1 diabetes (n = 86 853), inflammatory bowel disease (n = 48 073), spina bifida (n = 7819), and sickle cell anemia (n = 57 622) from January 1, 2014, to December 1, 2014.

**Exposures:**

Increasing age at index admission.

**Main Outcomes and Measures:**

Unplanned 30-day hospital readmission. Readmission odds were compared by patients’ ages in 2-year epochs (with age 15-16 years as the reference) using logistic regression, accounting for confounding patient characteristics and data clustering by hospital.

**Results:**

Of 215 580 participants, 115 982 (53.8%) were female; the median (interquartile range) age was 24 (20-27) years. Across CCDs, multimorbidity was common; the percentages of index hospitalizations with 4 or more coexisting conditions ranged from to 33.4% for inflammatory bowel disease to 74.2% for spina bifida. Thirty-day hospital readmission rates varied significantly across CCDs: 20.2% (cystic fibrosis), 19.8% (inflammatory bowel disease), 20.4% (spina bifida), 22.5% (type 1 diabetes), and 34.6% (sickle cell anemia). As age increased from 15 to 30 years, unadjusted, 30-day, unplanned hospital readmission rates increased significantly for all 5 CCD cohorts. In multivariable analysis, age trends in the adjusted odds of readmission varied across CCDs. For example, for AYA who had cystic fibrosis, the adjusted odds of readmission increased to 1.9 (95% CI, 1.5-2.3) by age 21 years and remained elevated through age 30 years. For AYA who had type 1 diabetes, the adjusted odds of readmission peaked at ages 23 to 24 years (odds ratio, 2.3; 95% CI, 2.1-2.6) and then declined through age 30 years.

**Conclusions and Relevance:**

These findings suggest that hospitalized AYA who have CCDs have high rates of multimorbidity and 30-day readmission. The adjusted odds of readmission for AYA varied significantly across CCDs with increasing age. Further attention is needed to hospital discharge care, self-management, and prevention of readmission in AYA with CCD.

## Key Points

**Question:**

How do readmission rates vary across complex chronic disease for adolescents and young adults with increasing age?

**Findings:**

In this cross-sectional study of 215 580 adolescents and young adults hospitalized for treatment of complex chronic diseases (cystic fibrosis, type 1 diabetes, inflammatory bowel disease, spina bifida, or sickle cell anemia), 30-day hospital readmission rates varied significantly across disease categories. As age increased from 15 to 30 years, unadjusted, 30-day, unplanned hospital readmission rates increased significantly for all complex chronic disease cohorts.

**Meaning:**

Further attention is needed to hospital discharge care, self-management, and prevention of readmission in adolescents and young adults with complex chronic disease.

## Teaser

This cross-sectional study assesses variation in 30-day unplanned hospital readmission rates among adolescents and young adults with complex chronic disease as they advance in age.

## Introduction

Adolescents and young adults (AYA) with complex chronic disease (CCD) are a growing population who experience severe, acute health problems.^1,2,3^ Examples of CCD include cystic fibrosis, type 1 diabetes, inflammatory bowel disease (IBD), sickle cell anemia, and spina bifida. Aging from adolescence to adulthood is an important process of AYA who have a CCD. As their age increases, many AYA who have a CCD may experience progression of disease, with new or worsening existing or coexisting illness and increased functional impairment.^4,5^ This fragile health status may lead to acute health problems, including infections and chronic condition exacerbations.

Hospitalization of AYA who have a CCD may be required for treatment of acute problems and for support during recovery from illness or procedures. Hospital discharge can be a vulnerable time for AYA who have a CCD, depending on their age and stage of transition to adulthood. For example, AYA with newly acquired autonomy and/or less familial support as they age may not fully appreciate the importance of medication adherence or discharge care plan instructions. Additionally, transitions of care in the outpatient setting to new adult-based practitioners may complicate hospital discharge plans, including contingency plans for problems that could arise. These hospital discharge experiences may vary by the type of CCD experienced by AYA. For example, AYA with CCDs that can rapidly worsen in severity with insufficient care management (eg, ketoacidosis with type 1 diabetes) may be more apt to follow through with discharge plans.

Emerging evidence suggests that age is a prominent risk factor associated with the odds of unplanned hospital readmission in AYA. One prior study^6^ reported a substantial increase in the odds of readmission for US adolescents as they aged into adulthood; their odds approximate the odds of readmission among elderly Medicare beneficiaries. As nationwide interest in reducing readmissions extends beyond elderly individuals, AYA—especially those with CCD—are an important, vulnerable population of hospitalized patients who deserve further attention.^7,8,9,10^ Therefore, we conducted the current national study to improve knowledge about hospitalized AYA who have a CCD and their readmissions during their transition to adulthood. For AYA aged 15 to 30 years, we assessed variation in the likelihood of readmission with increasing age and across different CCDs. We hypothesized that the likelihood of readmission would increase with increasing age and would vary significantly across CCDs.

## Methods

### Study Design and Setting

This is a retrospective 1-year cohort analysis of the 2014 Agency for Healthcare Research and Quality (AHRQ) Nationwide Readmissions Database (NRD), a database of all-payer hospital inpatient stays for patients of all ages. The NRD is drawn from 22 AHRQ State Inpatient Databases with variation in size and population density. The NRD contains verified patient identifiers to link individuals across hospitalizations. The 2014 NRD includes 14.9 million acute-care hospitalizations from 2048 hospitals, representing 35.3 million weighted total hospitalizations nationwide. This study, performed with a waiver of informed consent, was not considered human subjects research by the institutional review board at Boston Children’s Hospital because all data were deidentified. Presentation and discussion of study findings follow Strengthening the Reporting of Observational Studies in Epidemiology (STROBE) reporting guideline.

### Study Population

Index admissions (defined as any admission during the study period with 30-day eligibility for readmission measurement after discharge) for individuals aged 15 to 30 years at admission with cystic fibrosis, type 1 diabetes, IBD, spina bifida, and/or sickle cell anemia between January 1, 2014, and December 1, 2014, were identified using the AHRQ Chronic Condition Indicator and Clinical Classification System.^2,11^ These 5 diseases were chosen for analysis because each represents a distinct organ system, has associations with complex medical needs, and requires strong self-management skills to optimize well-being. We used the AHRQ Chronic Condition Indicator and Clinical Classification System *International Classification of Diseases, Ninth Revision, Clinical Modification* (*ICD-9-CM*) codes to distinguish the chronic diseases included in our study cohort. Index admission measurement ended on December 1, 2014, to allow for a full 30-day readmission period following all discharges. All-condition index admissions (ie, admission for any reason) were measured for individuals with the chronic diseases aside from admissions for pregnancy and childbirth, which were excluded using Major Diagnostic Category 14. Guided by methods used by the Centers for Medicare & Medicaid Services, we also excluded index admissions for patients who died, left against medical advice, were transferred to another acute-care hospital, or had a principal diagnosis of cancer (AHRQ Clinical Classification System groups 11-45).^12,13^

### Main Outcome Measure

The main outcome measure was 30-day, unplanned, all-cause readmission to any hospital following discharge from an index admission. Readmissions were defined using Centers for Medicare & Medicaid Services methodology.^13^ Centers for Medicare & Medicaid Services excludes planned readmissions using AHRQ’s Clinical Classification System, which uses principal *ICD-9-CM* diagnosis and procedure codes to identify admissions that are considered planned or potentially planned (eg, chemotherapy, labor and delivery).

### Index Admission Demographic and Clinical Characteristics

We assessed sex, payer (Medicare, Medicaid, private, self-pay, no charge, and other), length of stay, and discharge disposition (home with self-care, postacute care, home with home nursing services). We assessed the reason for each index admission using 3M Health Information System’s All Patient Refined Diagnosis Related Groups.^14,15^ We also assessed the quarter of the year for hospital discharge, with January through March composing the first quarter; April through June, the second; July through September, the third; and October through December, the fourth. Race/ethnicity was not assessed because it was not included in the NRD.

To count and describe patients’ coexisting chronic conditions, we used the AHRQ Chronic Condition Indicator,^11^ a tool that identifies conditions that are expected to last 12 months or longer and meet 1 or both of the following criteria: (1) the condition places limitations on self-care, independent living, and social interactions and (2) the condition results in the need for ongoing intervention with medical products, services, and special equipment.

### Hospital Characteristics

We assessed the teaching status and location of index and readmitting hospitals (metropolitan teaching, metropolitan nonteaching, and nonmetropolitan) as available in the NRD. A hospital was considered a teaching hospital if it had an American Medical Association–approved residency program, was a member of the Council of Teaching Hospitals, or had a 0.25 or higher ratio of full-time equivalent interns and residents to hospital beds. Metropolitan hospitals were those in small and large metropolitan areas defined by the US Department of Agriculture Urban Influence Codes.^16^

### Statistical Analysis

Using NRD weighting methods, we estimated the total number of index admissions and 30-day unplanned readmissions for all individuals aged 15 to 30 years at admission with each of the 5 CCD cohorts. The weighing method estimates findings from the NRD sample (n = 2006 hospitals) to the universe of all US hospitals and their discharges.^17^ We calculated discharge weights using poststratification on the sample hospital characteristics (US Census region, urban or rural location, teaching status, bed size, and hospital control) and patient’s sex and age. The target universe of inpatient discharges across all hospitals in the United States was determined for each stratum (defined by the hospital and patient characteristics listed) using AHRQ’s 47 State Inpatient Databases, which include 95% of all US hospital discharges, and American Hospital Association hospital discharge counts for hospitals not reported in the State Inpatient Databases. Within each stratum, each NRD inpatient admission received a discharge weight that was equal to the total number of US inpatient discharges it represented.

In bivariable analysis, comparisons of index admission characteristics between patients with and without a readmission were made using χ^2^ tests for categorical variables and Wilcoxon rank sum tests for continuous variables. In multivariable analysis, a logistic regression model was derived for individuals with each chronic disease (5 total models) to estimate the adjusted odds of readmission for age at admission using fixed effects to control for confounding variables known to influence the odds of readmission, including the number of chronic conditions, sex, payer, length of stay, discharge disposition, and hospital type.^14,18,19,20^ In the model, age was entered in 2-year increments with age 15 to 16 years as the reference. All analyses were performed using SAS statistical software version 9.4 (SAS Institute). Two-sided *P* values less than .001 were considered statistically significant owing to the large sample size.

## Results

### Characteristics of the Study Population

There were 215 580 all-cause index admissions (115 982 [53.8%] female; median [interquartile range] age, 24 [20-27] years) for individuals aged 15 to 30 years with cystic fibrosis (n = 15 213), type 1 diabetes (n = 86 853), IBD (n = 48 073), spina bifida (n = 7819), and sickle cell anemia (n = 57 622) (Table 1). There was a female predominance for all chronic diseases; this finding persisted when excluding admissions for pregnancy and childbirth. There was significant variation in Medicaid enrollment across disease categories (27.1% for IBD, 33.1% for cystic fibrosis, 41.6% for type 1 diabetes, 47.7% for spina bifida, and 58.5% for sickle cell anemia).

**Table 1.** Demographic and Hospitalization Characteristics of Individuals With Chronic Diseases Aged 15 to 30 Years at Admission

Characteristic	No. (%)				
	Type 1 Diabetes	Sickle Cell Disease	Inflammatory Bowel Disease	Cystic Fibrosis	Spina Bifida
Hospitalizations, No.	96 030	77 593	52 157	15 531	8404
Demographic characteristics					
Age at admission, median (IQR), y	23 (20-27)	24 (20-27)	24 (21-28)	22 (18-25)	23 (19-27)
Female	56 604 (58.9)	51 395 (66.2)	28 375 (54.4)	8562 (55.1)	5126 (61.0)
Payer					
Medicare	6868 (7.2)	11 219 (14.5)	2694 (5.2)	2010 (13.0)	1539 (18.4)
Medicaid	39 831 (41.6)	45 309 (58.5)	14 087 (27.1)	5114 (33.1)	4002 (47.7)
Private	32 092 (33.5)	15 200 (19.6)	28 799 (55.3)	7340 (47.5)	2440 (29.1)
Self-pay	11 666 (12.2)	2993 (3.9)	3495 (6.7)	267 (1.7)	150 (1.8)
No charge	1614 (1.7)	365 (0.5)	708 (1.4)	31 (0.2)	13 (0.2)
Other	3690 (3.9)	2324 (3.0)	2272 (4.4)	698 (4.5)	242 (2.9)
Hospitalization characteristics					
Length of stay, median (IQR), d	2 (2-4)	3 (2-6)	3 (2-6)	7 (4-13)	4 (2-7)
Type of index hospital					
Urban, teaching	62 032 (64.6)	61 239 (78.9)	39 787 (76.3)	14 360 (92.5)	6653 (79.2)
Urban, nonteaching	23 545 (24.5)	12 887 (16.6)	9805 (18.8)	823 (5.3)	1214 (14.4)
Rural	10 453 (10.9)	3467 (4.5)	2566 (4.9)	348 (2.2)	537 (6.4)
Discharge disposition					
In-hospital mortality	273 (0.3)	152 (0.2)	90 (0.2)	214 (1.3)	85 (1.0)
Routine to home	90 783 (94.6)	75 336 (97.1)	47 812 (91.7)	12 356 (79.6)	6439 (76.6)
Home health care	3644 (3.8)	1725 (2.2)	3826 (7.3)	3042 (19.6)	1425 (16.9)
Postacute care	1552 (1.6)	481 (0.6)	476 (0.9)	132 (0.8)	542 (6.4)

Abbreviation: IQR, interquartile range.

Regarding clinical characteristics, most hospitalized individuals had multiple coexisting conditions and many were assisted with medical technology (Table 2). The percentages of hospitalized AYA with 4 or more coexisting conditions were 33.4% for IBD, 34.2% for sickle cell anemia, 44.5% for diabetes, 65.2% for cystic fibrosis, and 74.2% for spina bifida. Among all 5 studied diseases, depression was one of the most common coexisting conditions (18.8% for sickle cell anemia, 21.0% for IBD, 24.0% for spina bifida, 28.4% for diabetes, and 34.2% for cystic fibrosis). There was significant variation across diseases in the percentages of individuals with technology assistance (3.1% of patients with sickle cell anemia; 5.0%, type 1 diabetes; 10.3%, IBD; 19.5%, cystic fibrosis; and 53.4%, spina bifida).

**Table 2.** Clinical Characteristics of Hospitalized Individuals With Chronic Diseases Aged 15 to 30 Years at Admission

Characteristic	No. (%)				
	Type 1 Diabetes	Sickle Cell Disease	Inflammatory Bowel Disease	Cystic Fibrosis	Spina Bifida
Hospitalizations, No.	96 030	77 593	52 157	15 531	8404
Technology assistance^a^	4772 (5.0)	2407 (3.1)	5372 (10.3)	3035 (19.5)	4486 (53.4)
Chronic conditions, No.^b^					
2-3	39 429 (41.1)	41 684 (53.7)	22 985 (44.1)	4542 (29.2)	1938 (23.1)
4-5	23 639 (24.6)	16 083 (20.7)	10 615 (20.4)	5155 (33.2)	2419 (28.8)
6-7	10 796 (11.2)	5025 (6.5)	4027 (7.7)	2956 (19)	1742 (20.7)
8-9	4577 (4.8)	1469 (1.9)	1457 (2.8)	1304 (8.4)	1007 (12)
≥10	2660 (2.8)	714 (0.9)	512 (1.0)	616 (4.0)	810 (9.6)
Most common coexisting chronic conditions (%)^b^					
No. 1	Depression (26.9)^c^	Asthma (26.7)	Anxiety (22.7)^c^	Type 2 diabetes (52.0)	Neurogenic bladder (52.1)
No. 2	Anxiety (20.9)^c^	Chronic pain (22.6)	Depression (20.2)^c^	Esophageal reflux (42.6)	Shunted hydrocephalus (41.7)
No. 3	Hypertension (19.8)	Aseptic necrosis hip (16.4)	Esophageal reflux (15.6)	Depression (33.8)^c^	Decubitus ulcer (25.2)
No. 4	Substance abuse (19.0)^c^	Depression (15.6)^c^	Asthma (12.0)	Malnutrition (28.9)	Paraplegia (24.2)
No. 5	Peripheral nerve disorder (17.4)	Hemochromatosis (13.9)	Chronic pain (11.7)	Anxiety (27.4)^c^	Depression (23.0)^c^

^a^ Technology assistance includes use of items such as gastrostomy, tracheostomy, cerebrospinal fluid shunt, insulin pump, and renal dialysis catheter.

^b^ Chronic conditions were distinguishing using the Agency for Healthcare Research and Quality Chronic Condition Indicator and Clinical Classification System.

^c^ Indicates coexisting conditions related to mental health and substance abuse.

### Index Admissions

Exacerbation of the underlying chronic disease was the most common reason for index admission for AYA with IBD (35.1%), sickle cell anemia (78.0%), diabetes (55.1%), and cystic fibrosis (60.9%). Participants with spina bifida had more diverse reasons for index admission, the most common of which was kidney and urinary tract infection (11.3%).

### 30-Day Unplanned Hospital Readmissions

**Figure 1.**
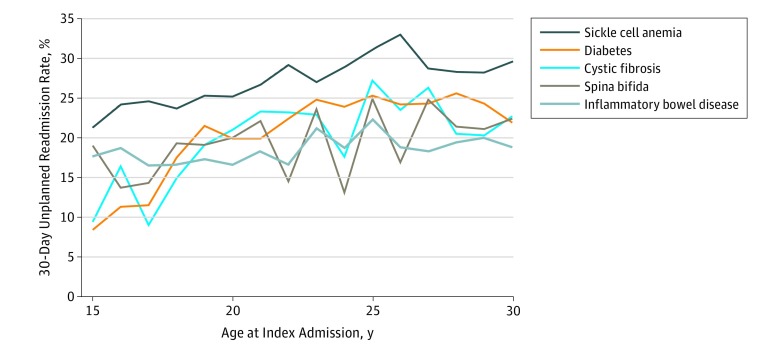
Trends in Unadjusted, 30-Day, Unplanned All-Cause Readmission Rates by Age at Admission The graph shows rates of 30-day, unplanned hospital readmission after index hospitalization for individuals with 5 chronic diseases. The rates are presented with increasing age in years.

Hospital readmission rates varied among the studied diseases (19.8% for IBD, 20.2% for cystic fibrosis, 20.4% for spina bifida, 22.5% for diabetes, and 34.6% for sickle cell anemia) and increased with increasing age (Figure 1). The smallest increase in readmission rate (31.3%) was observed with IBD (readmission rate, 17.1% at age 15 years to 21.8% at 30 years). The largest increase (138.7%) occurred with cystic fibrosis (8.2% at age 15 years to 29.0% at 30 years) (Figure 1). Readmission rate did not vary by season of discharge from the index admission.

Most reasons for the index hospitalization and readmission were directly related to the underlying primary chronic disease (Table 3). For example, 73.2% of index hospitalizations and 60.9% of readmissions for AYA with cystic fibrosis were for a cystic fibrosis exacerbation. Most readmissions (72.4%) occurred in the same hospital as the index admission. However, this percentage varied by disease (15.6% of AYA who had cystic fibrosis were readmitted to a different hospital compared with 23.8% with spina bifida, 24.3% with IBD, 25.6% with sickle cell anemia, and 29.1% with diabetes) (Table 3).

**Table 3.** Characteristics of 30-Day Unplanned Hospital Readmission for Individuals With Chronic Diseases Aged 15 to 30 Years at Index Admission

Characteristic	No. (%)				
	Type 1 Diabetes	Sickle Cell Disease	Inflammatory Bowel Disease	Cystic Fibrosis	Spina Bifida
30-d unplanned readmission rate, %	22.5	34.6	19.8	20.2	20.4
30-d readmissions, No.					
1	6907 (64.5)	4554 (53.4)	4869 (72.5)	951 (59.0)	719 (70.4)
2	1803 (16.8)	1630 (19.1)	1189 (17.7)	342 (21.2)	175 (17.2)
≥3	1998 (18.7)	2340 (27.5)	661 (9.8)	319 (19.8)	127 (12.5)
Time to readmission, median (IQR), d	12 (5-20)	14 (6-22)	11 (5-19)	15 (8-22)	12 (6-21)
Readmissions to different hospital, %	29.1	25.6	24.3	15.6	23.8
In-hospital mortality	76 (0.4)	73 (0.3)	44 (0.4)	82 (2.6)	14 (0.9)
Most common reasons for readmission (%)^a^					
No. 1	Diabetes (55.1)	Sickle cell anemia crisis (78)	Inflammatory bowel disease (35.1)	Cystic fibrosis (60.9)	Kidney and urinary tract infections (15.2)
No. 2	Peripheral, cranial and autonomic nerve disorders (9.8)	Other anemia and disorders of blood and blood-forming organs (2.5)	Major small and large bowel procedures (8.6)	Septicemia and disseminated infections (4.8)	Septicemia and disseminated infections (9)
No. 3	Septicemia and disseminated infections (2.8)	Pneumonia (1.6)	Major gastrointestinal and peritoneal infections (4.8)	Pancreatic disorder (3.8)	Malfunction, reaction, and complications of genitourinary device or procedure (5.1)
No. 4	Renal failure (2)	Septicemia and disseminated infections (1.5)	Intestinal obstruction (3.5)	Other digestive system diagnoses (2.2)	Ventricular shunt procedures (4.3)
No. 5	Pancreatic disorder (1.4)	Postoperative, posttraumatic, and other device infections (1.1)	Septicemia and disseminated infections (3.2)	Intestinal obstruction (2)	Postoperative, posttraumatic, and other device infections (3.8)

Abbreviation: IQR, interquartile range.

^a^ Reasons for readmission were distinguished 3M Health System’s All Patients Refined Diagnosis Related Groups.

### Multivariable Analysis of Patient Characteristics and 30-Day Unplanned Hospital Readmission

After controlling for patients’ other demographic and clinical characteristics, use of public insurance was associated with higher odds of readmission for all studied diseases; odds ratios (ORs) for readmission in AYA with public vs other types of insurance ranged from 1.2 (95% CI, 1.0-1.3) for spina bifida to 2.0 (95% CI, 1.9-2.1) for diabetes (eTable in the Supplement). For all diseases, higher odds of readmission were associated with higher number of coexisting chronic conditions. For instance, compared with AYA with cystic fibrosis and 1 coexisting chronic condition, the odds of readmission were 6 times higher for AYA with more than 8 coexisting conditions (OR, 6.2; 95% CI, 4.6-8.5) (eTable in the Supplement).

**Figure 2.**
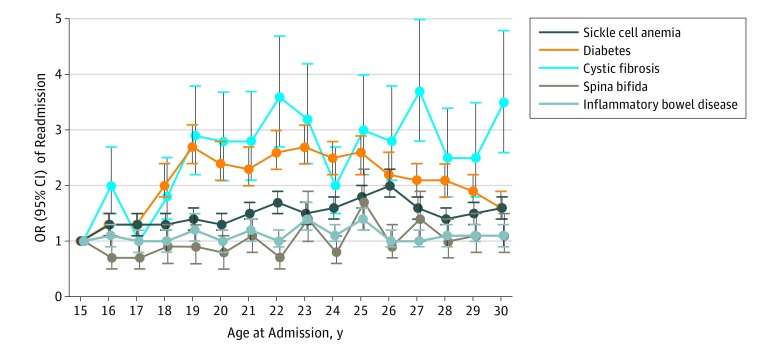
Adjusted Odds Ratios (ORs) of 30-Day Unplanned Hospital Readmission by Age at Index Admission The graph shows ORs with 95% confidence intervals of 30-day, unplanned hospital readmission by age in 2-year epochs. The ORs were adjusted for age, sex, payer, and number of chronic conditions.

There was variation across diseases in the association between increasing age and the adjusted odds of readmission (Figure 2). For example, the odds of readmission for AYA with cystic fibrosis were nearly 2 times higher (OR, 1.9; 95% CI, 1.6-2.3) for individuals aged 29 to 30 years vs 15 to 16 years. In contrast, only modest changes were observed in the odds of readmission for AYA ages 29 to 30 years vs 15 to 16 years with IBD (OR, 1.2; 95% CI, 1.0-1.3) and spina bifida (OR, 1.3; 95% CI, 1.1-1.7). For diabetes and sickle cell anemia, the adjusted odds of readmission peaked at ages 23 years (OR, 2.3; 95% CI, 2.1-2.6) and 26 years (OR, 2.0; 95% CI, 1.9-2.2), respectively. Unique among CCDs studied, patients with diabetes demonstrated an initial increase and subsequent decrease in the adjusted odds of readmission between ages 15 and 30 years (Figure 2).

## Discussion

This article describes the characteristics of hospitalized AYA who have a CCD and shows that this population experienced high rates of 30-day unplanned hospital readmission that increased with age. The hospitalized population of AYA who have CCDs is remarkable for its female predominance and its prevalence of comorbid conditions, particularly mental health diagnoses. Of the CCDs studied here, AYA who have sickle cell anemia had the highest unadjusted readmission rates. Adolescents and young adults who have cystic fibrosis experienced the largest increase in the adjusted odds of hospital readmission with increasing age. In contrast, AYA who have diabetes experienced an increase in the odds of readmission—peaking at 23 years—and a subsequent decrease with age. Across all CCDs, public insurance and multiple coexisting conditions were associated with higher odds of readmission.

Regarding patient characteristics, hospitalized AYA who have CCDs were predominantly female, with female patients accounting for three-fifths of admissions. Prior studies^21,22,23,24,25^ also report a predominance of female admissions across the CCDs studied. Sex differences in incidence of the studied CCDs do not explain this finding. For example, type 1 diabetes has a male predominance of indicence.^26^ Reproductive health care needs might be hypothesized to help explain this finding. However, admissions for pregnancy and childbirth were excluded from our analysis. Although not assessed in the current study, the female predominance in hospitalization may be related to prior investigations that show potential differences in self-management (eg, medication management),^27,28^ use of outpatient care (eg, for chronic disease management),^29,30^ disparate severity of disease (eg, earlier mortality in women with cystic fibrosis),^22,31^ and perceptions of symptoms that influence the decision to seek and provide inpatient care.^32^ Further investigation is thus needed to understand the causes and significance of the sex differences in hospital use for AYA who have CCDs.

In addition to the finding on sex, our study revealed a high prevalence of coexisting conditions in hospitalized AYA who have a CCD. Increased number of coexisting conditions was associated with higher odds of readmission. Because our cohort comprised hospitalized patients only, the high prevalence of the conditions may indicate a selection of high-risk patients with a more severe phenotype of disease. Many coexisting conditions were related to the pathophysiology of the underlying CCD. For example, patients with sickle cell anemia had a high prevalence of aseptic necrosis of the hip and patients with cystic fibrosis had a high prevalence of type 2 diabetes. Those types of coexisting conditions increase the complexity of care requirements (eg, additional monitoring, therapeutics). Thus, multimorbidity may represent an important crosscutting phenotype of AYA who have CCDs to target for high-quality discharge planning.

Relatedly, our study also found a high prevalence of depression in hospitalized AYA who have CCDs. Mental health diagnoses may compound the difficulty of CCD management by impairing patients’ abilities to adhere to discharge treatment plans. For example, in patients with type 1 diabetes, depression is associated with higher values of glycated hemoglobin.^33^ Prior studies also report an association between reduced lung function and depression in individuals with cystic fibrosis.^12,30,34,35^ Depression has also been associated with impaired disease control for IBD.^36^ It remains difficult to distinguish whether coexisting depression leads to worsened health or whether depression develops as a result of worsened health.^37^ Regardless, emerging mental health interventions for individuals with chronic disease show promise to optimize disease control.^36,38^ For example, intensive psychotherapy has been shown to decrease admissions for diabetic ketoacidosis in patients with diabetes.^38^ Insufficient access to and inconsistent use of these interventions complicate this proposition, especially during discharge planning.^39,40^ Therefore, the presence of a mental health diagnosis may be another indicator of the need for enhanced discharge planning for AYA who have a CCD.

Nearly one-fourth of hospitalized AYA who have a CCD in the current study experienced 30-day hospital readmission. This rate is 3-fold higher than the general population in this age group and also higher than Medicare beneficiaries older than 65 years.^6^ Among the AYA in the current study, those with sickle cell anemia demonstrated the highest 30-day readmission rate (28.1%). This high rate is consistent with prior studies; several attributes may explain it.^15,41,42^ Exacerbations of sickle cell anemia (eg, vaso-occlusive episodes) have high morbidity and often a rapid onset with severe health deterioration.^15,43^ Moreover, individuals with sickle cell anemia commonly have chronic exposures to pain medications, including opioids. These issues complicate intervention and management of sickle cell anemia exacerbations in the ambulatory setting. In addition, as we observed, individuals with sickle cell anemia use public insurance more than young adults with other CCDs. Adolescents and young adults with public insurance may also have social and financial challenges that could affect access to high-quality outpatient preventive and urgent care.^44,45^ The emergence of effective interventions to prevent and manage sickle cell episodes (eg, hydroxyurea and sickle cell day treatment programs) call attention to the need for safe discharge of hospitalized individuals with sickle cell anemia.

The contrasting trends in adjusted odds of readmissions with increasing age for AYA who have cystic fibrosis and diabetes merit discussion. While odds of readmission increased for patients with cystic fibrosis with increasing age, a similar initial increase in odds for patients with diabetes with increasing age was followed with a decrease as patients approached age 30 years. This difference is surprising and its cause is not obvious. Care management for both diseases is routinely associated with receipt of time-sensitive medications, use of durable medical equipment, and interpretation of laboratory and testing results (eg, blood glucose for diabetes; peak expiratory flow for cystic fibrosis), all of which may be required multiple times per day.^46,47^ Although the current study was not positioned to assess self-management of chronic disease, it is possible that increases in autonomy for AYA could have contributed to variable adherence to management strategies following discharge, resulting in increased likelihood of readmission during the adolescent years and early 20s.^38,48,49,50^ Relatedly, we speculate that the decrease in odds of readmission for AYA with diabetes from age 23 to 30 years could potentially reflect gains in self-management skills that stabilized and controlled diabetes severity. In contrast, hospitalized AYA with cystic fibrosis may have experienced increasing severity of disease with increasing age despite their best self-management efforts, which led to a steady increase in odds of readmission. Further investigation is necessary to assess those speculations—including how they might generalize to other CCDs—and how much emerging transition program interventions can help optimize self-care practices and stabilize health for AYA who have cystic fibrosis, in particular, soon after discharge.^34,46^

### Limitations

This study has several limitations. The cross-sectional design cannot make inferences about the health trajectory of individual AYAs as their ages increased from 15 to 30 years. Limitations also include data not available in the NRD. We were unable to assess a full calendar year of index admissions because January 30-day readmissions for December index admissions are not available in the database. Data on readmissions to hospitals in a different US state from the index admission hospital are not available in the NRD. Furthermore, the NRD does not contain data about characteristics such as functional status, self-management, social and familial issues, outpatient health care use, quality of life, and other characteristics associated with hospital readmissions. In addition, the NRD does not contain information on reason for insurance enrollment. Therefore, we were unable, for example, to assess how much Medicare enrollment in AYA was due to end-stage renal disease vs other enrollment qualifications (eg, being an adult dependent of a Medicare beneficiary).^51,52^

Reliance on diagnostic coding created additional limitations. The inpatient diagnosis billing codes did not convey information about the severity of the coexisting chronic conditions. Furthermore, it is possible that some conditions may be undercoded in the inpatient setting. The NRD does not contain data on inpatient clinicians, including their training and field. Therefore, we could neither cluster data by clinician nor assess whether pediatric or adult practitioners discharged the AYA.

## Conclusions

The examinations from the current study underscore the vulnerability of health after discharge for AYA who have a CCD. Adolescents and young adults who have a CCD have 30-day unplanned hospital readmission rates that increase with age and that are 3 times higher than the general population of AYA. Increased attention to hospital readmissions in AYA who have a CCD is necessary to optimize their health and safety at hospital discharge. Future studies should assess how self-management of CCD and transfer of care to adult health care practitioners influences the likelihood of hospital readmission. Future investigations might also include CCDs beyond the 5 we selected such that overarching trends in readmission rates across age can be identified.

In the interim, hospital and outpatient practitioners may find the results from the current study useful during discharge planning. Helping AYA who have a CCD understand the elevated risk of hospital readmission and how this risk increases with age may be important. Paying particular attention to processes of discharge care that AYA might experience differently in context of pediatric vs adult care may be important; examples of such processes include (1) assessing readiness for hospital discharge, (2) creating contingency plans for problems that might arise after discharge, and (3) making appointments for follow-up with outpatient and community practitioners after discharge. Those discharge care activities might be especially pertinent for particular types of AYA, including those with multimorbidity and mental health diagnoses.

## Notes

Supplement. **eTable.** Multivariable Analysis of Risk Factors for 30-Day Unplanned Hospital Readmission for Individuals With Chronic Diseases Age 15-to-30 Years at Index Admission
