# Supplementary material for: Hospital Readmission of Adolescents and Young Adults With Complex Chronic Disease
Source: JAMA Netw Open. 2019 Jul 24;2(7):e197613. doi: 10.1001/jamanetworkopen.2019.7613 (PMC6659144; doi:10.1001/jamanetworkopen.2019.7613)
Supplement: Supplement. — eTable. Multivariable Analysis of Risk Factors for 30-Day Unplanned Hospital Readmission for Individuals With Chronic Diseases Age 15-to-30 Years at Index Admission [file jamanetwopen-2-e197613-s001.pdf]

Supplementary Online Content

Dunbar P, Hall M, Gay JC, et al. Hospital readmission of adolescents and young adults with complex chronic disease. *JAMA Netw Open*. 2019;2(7):e197613. doi:10.1001/jamanetworkopen.2019.7613

eTable. Multivariable Analysis of Risk Factors for 30-Day Unplanned Hospital Readmission for Individuals With Chronic Diseases Age 15-to-30 Years at Index Admission

This supplementary material has been provided by the authors to give readers additional information about their work.

© 2019 Dunbar P et al. *JAMA Network Open.*

eTable. Multivariable Analysis of Risk Factors for 30-day Unplanned Hospital Readmission for Individuals with Chronic Diseases Age 15-to-30 Years at Index Admission.

Characteristic	Adjusted Odds Ratio (95% Confidence Interval) of Hospital Readmission[1]				
	**Type 1 Diabetes**	**Sickle cell anemia**	**Inf. Bowel Disease**	**Cystic fibrosis**	**Spina bifida**
**Sex** (ref = male)					
Female	1.3 (1.3, 1.4)	0.7 (0.7, 0.7)	0.9 (0.9, 1)	1.2 (1.1, 1.3)	1.6 (1.4, 1.8)
**Payor** (ref = private)					
Medicare	2.4 (2.2, 2.5)	2.3 (2.1, 2.4)	2.1 (1.9, 2.3)	1.3 (1.1, 1.5)	1.3 (1.1, 1.5)
Medicaid	2 (1.9, 2)	1.5 (1.5, 1.6)	1.4 (1.3, 1.5)	1.3 (1.2, 1.4)	1.2 (1, 1.3)
Self pay	1.4 (1.3, 1.5)	0.9 (0.8, 1)	1.1 (1, 1.2)	1.5 (1.1, 2.1)	1.6 (1.1, 2.4)
No charge	1.9 (1.7, 2.1)	1.1 (0.8, 1.4)	1.8 (1.5, 2.1)	NA	0.7 (0.1, 3.8)
Other	1.3 (1.2, 1.4)	1.9 (1.7, 2.1)	1.2 (1.1, 1.4)	2 (1.7, 2.4)	0.8 (0.5, 1.2)
**Patient Residence** (ref = metropolitan)					
Rural	1.1 (1, 1.1)	1.3 (1.2, 1.4)	1.1 (1, 1.2)	0.9 (0.8, 1.1)	0.6 (0.4, 0.7)
Micropolitan	1 (0.9, 1.1)	0.9 (0.8, 1)	1.1 (1, 1.2)	1.1 (1, 1.3)	0.6 (0.5, 0.8)
Suburbian	1 (0.9, 1)	0.9 (0.8, 0.9)	1 (0.9, 1)	1.2 (1.1, 1.3)	0.7 (0.7, 0.8)
**Chronic Conditions of Any Complexity** (ref = 1 chronic condition)					
10+	3.4 (3, 3.8)	1 (0.8, 1.2)	1.6 (1.3, 2)	4.5 (3.2, 6.4)	2.8 (1.9, 4.1)
8-9	2.9 (2.7, 3.2)	1.3 (1.1, 1.5)	1.8 (1.6, 2.1)	5.8 (4.2, 7.9)	1.9 (1.3, 2.7)
6-7	2.6 (2.4, 2.8)	1.2 (1.1, 1.2)	1.8 (1.6, 2)	4.4 (3.3, 6)	1.5 (1.1, 2.1)
4-5	1.9 (1.8, 2)	1 (1, 1.1)	1.5 (1.4, 1.6)	3.8 (2.8, 5.1)	1.7 (1.2, 2.3)
2-3	1.3 (1.2, 1.4)	0.8 (0.7, 0.8)	1.2 (1.1, 1.2)	2.6 (2, 3.5)	1.3 (0.9, 1.8)
**Complex Chronic Conditions** (ref = the absence of each complex chronic condition[2])					
Neuromuscular	0.7 (0.6, 0.8)	0.8 (0.7, 0.9)	0.7 (0.6, 0.9)	0.8 (0.6, 1.1)	NA
Cardiovascular	0.9 (0.9, 1)	1 (1, 1.1)	1.1 (1, 1.2)	1.3 (1.1, 1.6)	0.7 (0.6, 0.9)
Respiratory	1.1 (0.9, 1.3)	1.4 (1.1, 1.7)	1 (0.8, 1.3)	NA	0.9 (0.7, 1.2)
Renal	1.6 (1.5, 1.7)	1.2 (1.1, 1.3)	1.4 (1.2, 1.6)	0.7 (0.6, 0.9)	1.4 (1.2, 1.6)

© 2019 Dunbar P et al. *JAMA Network Open.*

Gastrointestinal	1.2 (1.1, 1.3)	0.9 (0.8, 1)	NA	1 (0.8, 1.1)	1.4 (1.2, 1.6)
Hematology and immunodeficiency	1.1 (1, 1.2)	NA	1 (0.9, 1.1)	1.5 (1.2, 1.8)	1 (0.7, 1.5)
Metabolic	1 (1, 1)	1.5 (1.5, 1.6)	1 (0.9, 1.1)	1 (0.9, 1.1)	1.1 (0.9, 1.3)
Congenital or genetic defect	0.9 (0.8, 1.1)	0.9 (0.7, 1)	0.8 (0.7, 0.9)	0.8 (0.6, 1)	0.8 (0.7, 0.9)
Malignancy	1.1 (1, 1.3)	1.2 (1.1, 1.4)	1.4 (1.2, 1.5)	1.4 (1, 2.1)	1 (0.6, 1.7)
Technology Assistance	1.2 (1.1, 1.3)	1.2 (1.1, 1.3)	1.5 (1.4, 1.6)	1.4 (1.2, 1.6)	1.1 (1, 1.3)
Transplant	0.8 (0.7, 0.8)	1 (0.8, 1.3)	1.2 (0.9, 1.4)	1.6 (1.4, 1.9)	1.4 (0.9, 2)
**Disposition** (ref = to home, routine)					
Post-Acute Care[3]	0.8 (0.7, 0.9)	0.7 (0.6, 0.9)	1.1 (0.9, 1.4)	1.6 (1.1, 2.4)	0.7 (0.5, 0.9)
Home Health Care	1.2 (1.1, 1.2)	1.2 (1.1, 1.3)	1.5 (1.4, 1.6)	0.9 (0.8, 1)	1.5 (1.3, 1.7)

Abbreviations: ref = reference group; NA = not applicable

[1]Shown are the adjusted odds ratios for readmission for each complex chronic disease cohort. Five separate multivariable regression models were derived for each of the diseases.

[2]The adjusted odds ratios shown indicate the odds of hospital readmission in the presence vs. absence of each complex chronic condition. For example, in adolescents and young adults with type 1 diabetes, the presence of a renal complex condition was associated with an increased odds of readmission [1.6 (95%CI 1.5-1.7)].

[3]Includes Skilled Nursing Facility (SNF), Intermediate Care Facility (ICF), Another Type of Facility

© 2019 Dunbar P et al. *JAMA Network Open.*
